# Investigation of the cooperative-effects of Lewis- and Brønstedt acids in homogeneously catalyzed OME fuel synthesis by inline-NMR monitoring†

**DOI:** 10.1039/d4ra00744a

**Published:** 2024-05-08

**Authors:** Patrick Endres, Timo Schuett, Stefan Zechel, Martin D. Hager, Robert Geitner, Ulrich S. Schubert

**Affiliations:** a Laboratory of Organic and Macromolecular Chemistry (IOMC), Friedrich Schiller University Jena Humboldtstr. 10 07743 Jena Germany ulrich.schubert@uni-jena.de www.schubert-group.de; b Jena Center for Soft Matter (JCSM), Friedrich Schiller University Jena Philosophenweg 7 Germany; c Center of Energy and Environmental Chemistry Jena (CEEC Jena), Friedrich Schiller University Jena Philosophenweg 7a 07743 Jena Germany; d Helmholtz Insitute for Polymers in Energy Applications Jena (HIPOLE Jena), Friedrich Schiller University Jena Lessingstraße 12-14 07743 Jena Germany; e Institute for Chemistry and Bioengineering, Technical University Ilmenau Weimarer Str. 32 98693 Ilmenau Germany robert.geitner@tu-ilmenau.de

## Abstract

*Via* inline-nuclear magnetic resonance measurements, the homogeneously catalyzed poly(oxymethylene dimethyl ether) fuel synthesis using trioxane and dimethoxy methane is investigated. Besides the Brønsted acid (BA) catalyst triflic acid (TfOH) different metal halides are studied as Lewis-acidic (LA) catalysts. Among the used LAs, MgCl_2_, the weakest based on electronegativity, reveals the highest catalytical activity. Additionally, the influence of the concentration of BA and LA is investigated. An increase in BA concentration leads to an exponential increase of the reaction rate, while increasing the concentration of the LA leads to a volcano plot with its optimum at a LA : BA ratio of 1 : 3. The influence of the LA on the electron density of the intermediate formaldehyde is concluded as the main factor for this behavior.

## Abstract 2

The cooperative effect of Lewis and Brønsted acids within the OME fuel synthesis was investigated *via* inline NMR monitoring.

## Graphical abstract

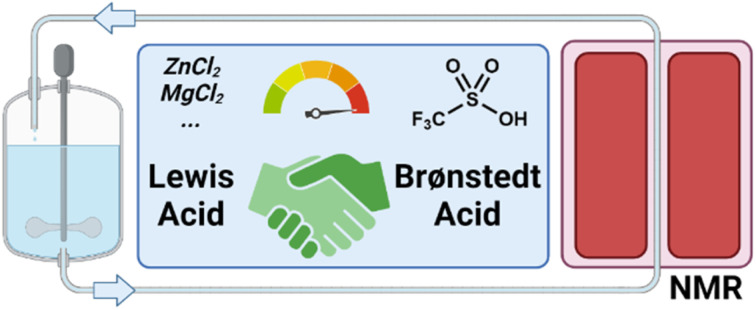

## Introduction

The potential of direct and indirect electrification on key greenhouse gas emitting sectors such as heat, industry or transportation are intensively discussed in the literature.^1–4^ Nevertheless, direct electrification is not automatically a suitable solution for every sector. Not just because of the technological challenges but also due to the limited local availability of renewable energy, which requires transport *via* energy carriers. In particular in the transportation sector, *e.g.*, for aviation,^5^ maritime transport^6^ and heavy duty vehicles,^7^ direct electrification remains challenging. In these fields, synthetic, carbon neutral fuels deliver a possible solution.^8^ The new type of fuel must meet different requirements. Besides being produced from renewable energy and a sustainable carbon source, the possibility of direct blending into conventional fuel is of great importance.^9^ This ensures that the synthetic fuel can be applied as drop in fuel within the existing infrastructure.

Regarding the group of synthetic fuels, Fischer–Tropsch-Fuels (FT-Fuels)^10–12^ and poly(oxymethylene dimethyl ether)s (OMEs or POMDEs)^13–16^ represent promising candidates. The first are chemically similar to their fossil counterparts, which enables the limitless blending. However, they also feature similar emission characteristics, which causes critical local pollutions. OME-based fuels cannot only be blended into existing fossil Diesel fuel but also enhances the combustion characteristics. OMEs are oxygenates without direct carbon–carbon bonds.^9,17^ This leads to a cleaner combustion and, therefore, soot reduction and lowering of particle size as well as matter. The preferred chain length of OME fuels for Diesel engines is between three and five repeating units (OME_3–5_).^18,19^

Their synthesis is based on formaldehyde and a methyl endcapping source. As a formaldehyde source, para-formaldehyde (pFA)^20^ or trioxane (TRI)^21^ are mostly applied in literature. As the latter, methanol^22^ and formaldehyde dimethylene acetal (DMM)^23^ are frequently utilized. By combining TRI and DMM a reactive system is generated in which no water formation occurs leading to a reduction of possible side reactions. Consequently, a cleaner product mixture is obtained, which facilitates not only the industrial scale OME-fuel production but also reaction monitoring. The synthesis itself is based on an acid catalyzed process.^17^ Therefore and due to the advantages of heterogeneous catalysis in other industrial applications, a variety of solid acids has been applied for the OME fuel synthesis.^17,22,24^ Mostly these acids are based either on zeolites^13,25–27^ or polymeric ion exchange resins.^28–30^ Nevertheless, the catalytic activity of a solid material is not just determined by the number of acidic sites, but also by the accessibility of these sites. Therefore, the surface structure, pore size, adsorption and desorption on the catalyst play an important role for its performance.^31–34^ Furthermore, the sampling process might influence the kinetic investigations due to a change of the reaction mixture to catalyst ratio.

On the contrary, in homogeneous catalysis, the activity is mainly reduced to the chemical properties of the catalytically active material. If homogeneous conditions are provided, the catalyst loading will not be influenced by the sampling process.

### Cooperative effect

Brønsted acids (BAs) and Lewis acids (LAs) are known for their catalytic activity during the OME fuel synthesis.^35,36^ Several studies already addressed the possible cooperative effects.^37,38^ Baranwoski *et al.* investigated the influence of the formaldeyhde content in dealuminated tin grafted zeolites. A synergistic effect was observed resulting in an increase of reaction rate in the presence of LA and BA sites within the zeolite structure. This effect was explained by additional activation of the formaldehyde (FA) unit by the LA site.^37^ Liu *et al.* also investigated the catalytic efficiency of sulfur doped titanium catalysts with different LA and BA contents.^38^ In this work a synergistic effect was observed whilst the LA revealed no activity as long as no BA was present. The influence of the heterogeneous nature was also discussed. One main problem is the subsequent or simultanous adsorption of the different starting materials on the catalyst surface. This process can strongly influence the reaction rate of the OME fuel synthesis. Furthermore, the total acid sites present on the catalytic surface influenced the reaction rate. To overcome this problem, the homogeneous synthesis of OME fuels needs to be further investigated.

In a recent study published by our groups, a new system to monitor the OME fuel synthesis *via* inline NMR measurements was introduced.^39^ This system is based on a benchtop NMR device, which already gained much interest in academic research.^40–43^ By the use of flow cells, a continuous circulation of the reaction solution through the analytical capillary is possible.^44^ Therefore, no sampling process is required and, thus, the spectroscopic technique does not affect the catalytic system. Consequently, inline NMR spectroscopy increases the time and chemical resolution while not influencing the catalytic reaction studied. Due to the combination of structural and quantitative information obtained by NMR spectroscopy as well as its decent measurement frequency, this technique is predestined for the monitoring of this reaction. Other possible techniques such as mass spectrometry or IR-spectroscopy bear several disadvantages. For example, these techniques are, in case of mass spectrometry, difficult to implement into an inline reaction setup and destroy the sample itself. Furthermore, they do not deliver detailed structural information for this kind of reaction (in particular for IR spectroscopy). IR spectroscopy is faster compared to NMR measurements, however, does not deliver suitable structural information to distinguish between the different OME species as the characteristic C–O and C–H stretching vibrations within the IR spectra are similar.^45^

Within the OME fuel reaction, first insights into the catalytical system were obtained by determining the order in catalyst of a liquid and solid acid.^39^

In the present study we follow up on our initial results and use the developed setup to study the highly debated cooperative effects of BAs and LAs during OME synthesis. Therefore, different LAs were utilized for the OME fuel synthesis and the influence of different LA : BA ratios on the catalytic performance was investigated using inline NMR spectroscopy.

## Results and discussion

### General aspects

**Scheme 1:**
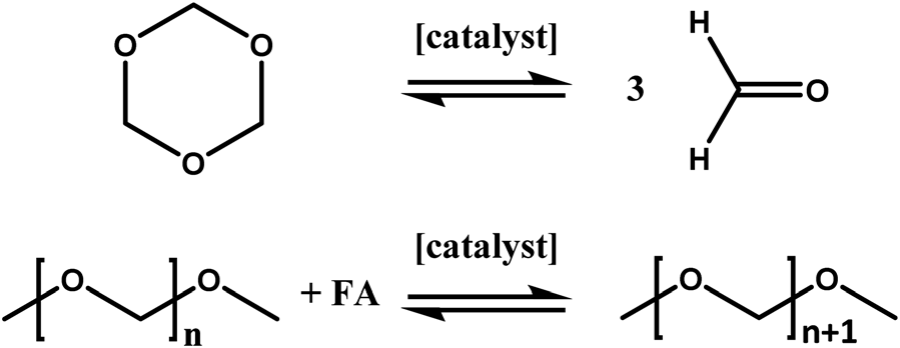
Schematic representation of the two intermediate steps involved in the OME synthesis *via* OME_1_ and TRI.

As mentioned above, inline NMR measurements were applied for the investigation of the catalytic system. The power of this setup is the possibility to investigate homogeneous reactions with only a short time delay and inline without the necessity of sample preparation. This eliminates possible errors occurring during the sampling process. Furthermore, the time required for the analysis of the reaction is reduced significantly due to the nature of the setup. An analysis by GC measurements could require about 45 minutes per single sample, which is almost the entire reaction time of the measurements performed within this study.^21^ Furthermore, extensive calibration and maintenance of the GC is necessary, compared to the low effort required for benchtop NMR devices.^23^ The anhydrous synthesis of OME fuels based on DMM and TRI was chosen not only due to its lower tendency to form byproducts but also to ensure the comparability with previous results.^39^ Within this synthesis a two-step reaction mechanism (Scheme 1) is widely assumed in literature.^17^ In the first step trioxane decomposition takes place resulting in the release of three formaldehyde (FA) molecules. In the second step, these FA units are reversibly incorporated into OME_1_ which results in a Schulz–Flory like distribution of OME_*n*_ upon multiple FA incorporation. As previously reported, more than one catalytic role of the catalyst is assumed, which will be addressed in this study by investigating the synergetic effects of Lewis- and Brønsted acids.^39^ In all experiments of this study in which a Lewis acid was utilized, the acid was added to the reaction medium directly after loading the reactor with TRI (44.4 g) and DMM (150 g). Afterwards, the reaction setup was tempered to 20 °C and the reaction medium was stirred to guarantee a homogeneous solution. Even though this progress took up to about 30 minutes, no turnover of the reaction was detected as long as no triflic acid (TfOH) was added. Corresponding spectra can be found in the ESI.† Consequently, it can be concluded that the presence of a BA is crucial for the reaction to proceed. This corresponds with results obtained in literature in which pure ZnCl_2_ showed almost no conversion at considerably higher temperatures.^46^

### Type of Lewis acid

The first part of the study aimed to screen different commercially available LAs to determine a suitable co-catalyst. For this purpose, FeCl_2_, ZnCl_2_, ZnBr_2_, AlCl_3_ and MgCl_2_ were utilized. The aim was to determine a reaction system in which the type of LA as well as a suitable concentration range of BA and LA fit to the analytical window and are not too slow to be measured within a reasonable time but also not too fast to be investigated in detail.

**Fig. 1:**
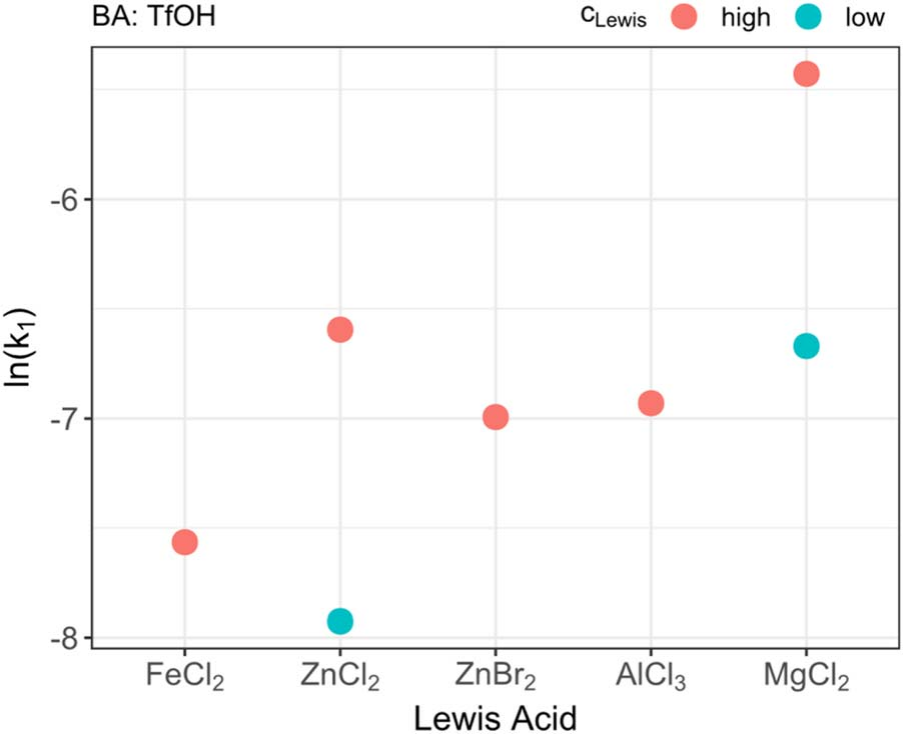
Catalytical performance of different Lewis acids as indicated by their ln(*k*_1_) value. *k*_1_ denotes the kinetic rate for the trioxane decomposition. For ZnCl_2_ and MgCl_2_ concentration-dependent measurements were performed with high and low concentrations. The corresponding values can be seen in Table 1.

**Scheme 2:**
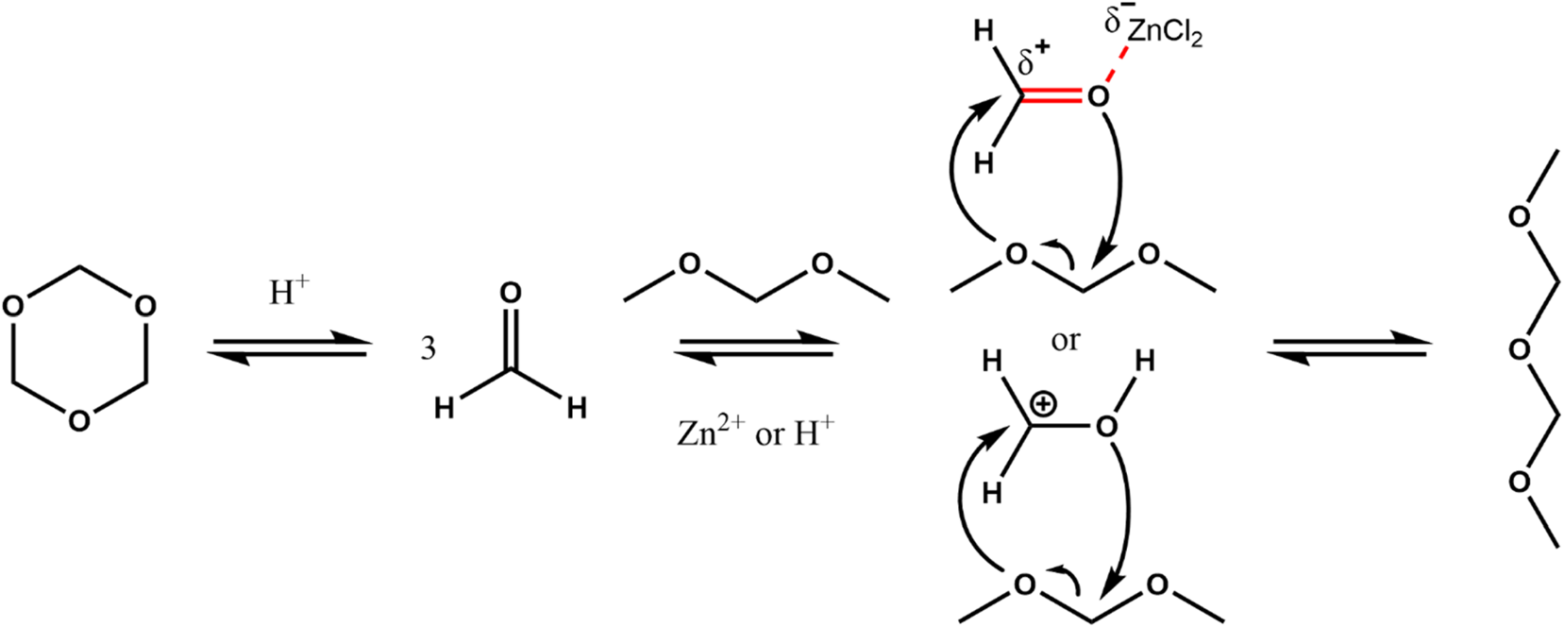
Schematic representation of a possible catalytic mechanism based on BA and LA catalysis.

As depicted in Fig. 1 there is a strong influence of the type of LA on the reaction behavior. A reason for this observation might be seen in the Lewis acidity of the used substances. LAs are substances which are capable of accepting additional electron pairs from other molecules.^47^ This ability is strongly influenced by the Pauling electronegativity of the respective ions involved in the catalytic process.^48^ This can be seen as a reason for the observed order in activity of the different acids. Hence, the order of the Lewis acidity of the different LAs used in this study is Fe(ii), Zn(ii), Al(iii), Mg(ii). The different efficiency between ZnCl_2_ and ZnBr_2_ can be attributed to the respective counterion. Chloride itself has a higher electron withdrawing effect towards the Zn(ii) ion. Thus, the electronegativity and, consequently, the Lewis acidity is increased. Although this principle might provide a suitable explanation, other factors such as the type of solvent might as well influence the Lewis acidity. Nevertheless, it can be estimated that the reactivity of the LAs is decreasing with increasing Lewis-acidity. A reason for this effect might be found in the electron density of the formaldehyde molecule which is influenced by the LA. The interaction of the LA with the oxygen atom of the formaldehyde results in a positive partial charge at the carbon atom and a reduced electron density at the oxygen atom. By the positive partial charge, the reaction is accelerated whereas the reduced electron density at the oxygen atom decreases the reaction rate. By further increasing the LA strength this effect is enhanced. Nevertheless, the reaction rate reducing effect at the oxygen atom is dominant and, thus, the reaction rate decreases with increasing LA strength. The catalytic mechanism based on the BA is consequently assumed as formation of a carbocation and an insertion of this intermediate species in the OME-chain. A possible reaction scheme with both catalytic roles which might explain these findings is depicted in Scheme 2. With the results on the type of LA in mind, MgCl_2_ and ZnCl_2_ were tested at two different concentrations. MgCl_2_ revealed a strong increase in reaction rate from *k* = 12.7 × 10^−4^ s^−1^ to *k* = 43.9 × 10^−4^ s^−1^ by doubling the amount of acid. For ZnCl_2_ the same from *k* = 3.6 × 10^−4^ s^−1^ to *k* = 13.7 × 10^−4^ s^−1^ was observed. To study the kinetic network underlying the BA–LA co-catalysis during OME production the reaction catalyzed by a high concentration of MgCl_2_ was too fast. Thus, it was chosen to conduct the following experiments with low ZnCl_2_ concentrations as LA.

### Concentration of the Brønsted acid

**Fig. 2:**
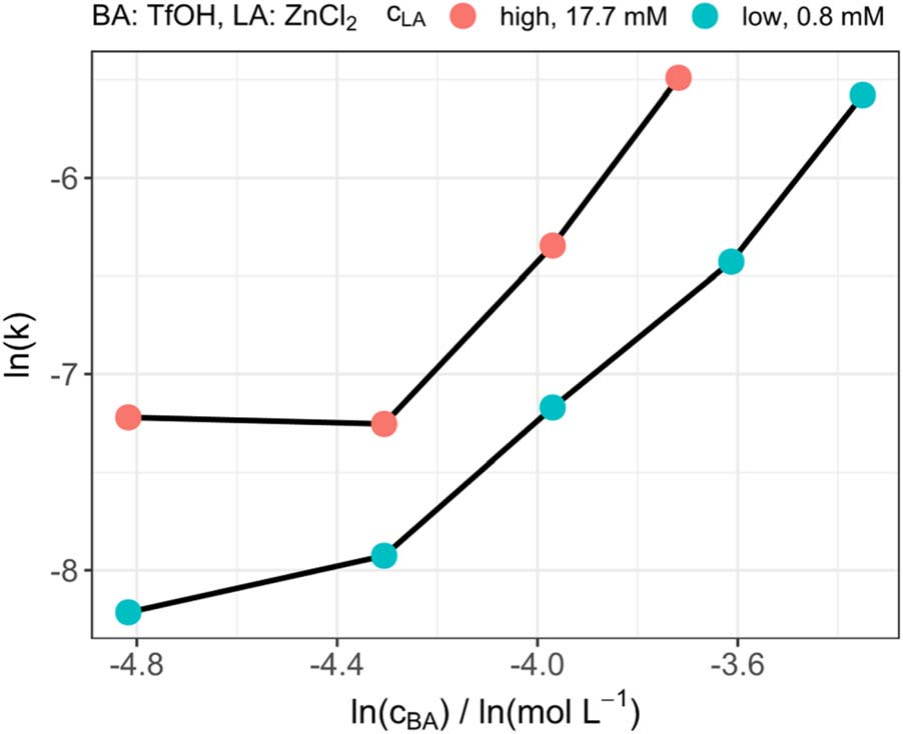
Catalytic performance of different amounts of Brønsted acid at two different Lewis acid concentrations.

After the selection of ZnCl_2_ as suitable LA for reaction monitoring, the influence of the amount of BA in the presence of the LA was investigated and compared to our previously reported results.^39^ As depicted in Fig. 2, when the LA concentration is kept constant, the reaction rate increased exponentially by increasing the amount of TfOH. Whereas, if the amount of LA was varied and the BA amount was kept constant, all reactions were accelerated in the same order of magnitude. The exponential increase supports our reported assumption of the homogeneous catalytic system not following a first order dependency in terms of the catalyst concentration.^39^ To explain these results an indirect or a direct influence of the LA on the reaction speed could be assumed. The LA could form a Lewis–Brønsted adduct with TfOH which then indirectly increases the Brønsted acid strength and, consequently, the reaction rate. The influence would therefore lead to an increased reaction rate of both intermediate reaction steps which are depicted in Scheme 1. Nevertheless, in this catalytic mechanism the reaction rate is directly dependent of the LA strength, which would not be in accordance with the aforementioned results. Therefore, the increase of the reaction speed by increasing the BA concentration might be caused by the additional formaldehyde which is provided by the enhanced TRI decomposition. The insertion of the FA into the OME is subsequently catalyzed by either the LA or BA, which is why the reaction speed increases almost uniformly by increasing the LA concentration. This supports the theory of the proposed multiple roles; the catalyst can play during the OME synthesis.

### Concentration of the Lewis acid

**Fig. 3:**
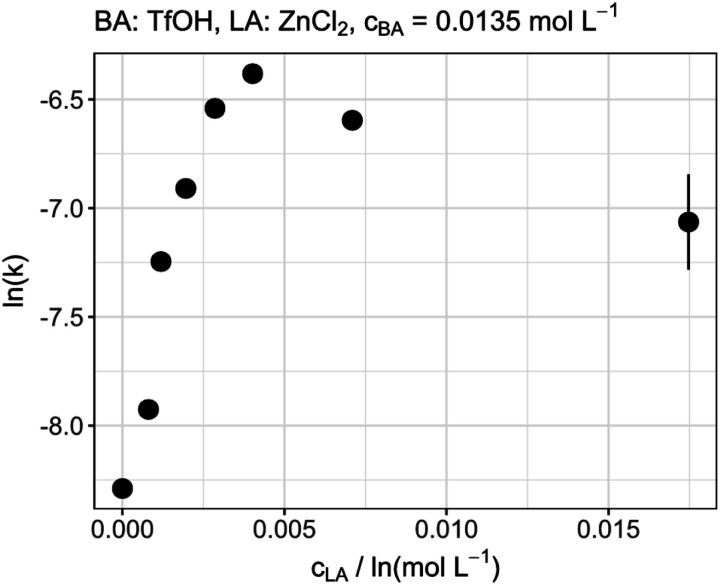
Catalytic performance of different Lewis acid amounts at constant Brønsted acid concentration.

The influence of the LA concentration was investigated by an experiment in which the amount of TfOH was kept constant and the amount of ZnCl_2_ was varied. Beginning at low concentrations the rate increased significantly up to a concentration of about 0.005 mol L^−1^, which corresponds to a ratio of about 1 : 3 (LA : BA). Interestingly, a further increase of the Lewis acid concentration did not result in a higher reaction rate but on the contrary decreased the reaction rate. This behavior can be seen in Fig. 3 as a volcano plot. These results do also not support the above-mentioned theory of the simple increase in BA strength based on the formation of a BA/LA adduct as a further increase of the reaction rate with an increasing LA concentration would have been expected. Although, the general presence of the effect cannot be excluded, a strong influence of it would also contradict the already described results regarding the influence of LA acid strength on the reaction rate. Therefore, it can be concluded that the LA can directly catalyze the incorporation of formaldehyde into the OME molecules. Nevertheless, a decrease of the reaction rate was observed by further increasing the LA concentration. This supports the theory of the electron density of the formaldehyde oxygen atom being crucial for the reaction rate. By increasing the LA concentration, the reaction of FA with OME to prolong the chain is accelerated. Consequently, the amount of free FA within the reaction is reduced. Therefore, the probability of multiple LA molecules interacting with one free FA is increasing. This, in turn, has a similar effect as the increase in LA strength as the electron density of the oxygen is further reduced leading to a slow down of the reaction rate.

## Experimental

All reactions and measurements as well as data evaluations were performed as previously described in literature.^39^

### Materials and methods

All chemicals were used as received from TCI, Sigma Aldrich and VWR, if not otherwise stated. Inline NMR spectra were recorded using a benchtop 43 MHz Spinsolve NMR spectrometer (Magritek, Germany) running on Spinsolve version 1.18.1. As advised by the manufacturer, the spectrometer was shimmed repeatedly using a solution of 90% D_2_O and 10% H_2_O. A glass capillary from the same manufacturer was inserted into the spectrometer to transfer the sample solution through the NMR spectrometer with a flow rate of 0.7 mL min^−1^. Every measurement sequence was performed in an infinite loop, starting with a shim on sample, followed by ten ^1^H NMR spectra, each in the form of a free induction decay (FID) that was recorded every minute with a pulse length of 8.6 μs, a total acquisition time of 15 s, an acquisition delay of 20 s and a dwell time of 200 μs. This results in 32 768 data points spread across an interval from −52.77 to 65.32 ppm.

The double jacket reactor was connected to a Huber Unistat Tango with a temperature range between −45 to 250 °C and an accuracy of ±0.01 °C. The thermostat was equipped with a Pt100 temperature sensor from the same supplier, monitoring the reaction temperature with a data point every five seconds. The reaction solution was pumped through the NMR spectrometer utilizing a LP-BT100-2J peristaltic pump from LongerPump equipped with a YZII15 pump head with three rollers. For adequate pumping, the 1/8′′ PTFE tubing was substituted at the pump with a Tygon S3 E-LFL tubing with an inner diameter of 4.8 mm and an outer diameter of 8.0 mm.

### Catalyst screening experiments

The tubing and the reactor were filled with a 20 mol% solution of trioxane (44.4 g, 0.49 mol) in formaldehyde dimethyl acetal (150 g, 1.97 mol). If a Lewis acidic catalyst was used, the respective amount (masses as stated in Table 1) was dissolved in the reaction solution. The jacket of the reactor and the reaction solution were heated or cooled to the respective temperature and the solution was pumped with a volume of 0.7 mL min^−1^ through the system for two minutes. Afterwards, the NMR spectrometer and the temperature monitoring were started and after an equilibration time of up to one hour the catalyst (masses as stated in Table 1) was added to the reactor. The difference between the temperature set in the Huber and measured in the reaction mixture was below 1 °C in all experiments. All reaction temperatures varied ±0.2 °C. The calculations are based on the measured temperature inside the reactor. The monitoring was stopped after no major changes of the reaction mixture were observed (min. 2 h). Single deviations of datapoints were caused by small gas bubbles entering the analytical cycle, which led to temporary broadening of the NMR signals.

**Table 1:** Overview of all reactions performed and calculated *k*_1_ values; temperature (20 °C), amount of DMM (150 g) and trioxane (44.4 g) were constant

Exp.	*c*(TfOH) [mM]	Lewis acid	*c*(Lewis acid) [mM]	*k*_0_[10^−4^ s^−1^]
1	13.5	ZnCl_2_	2.9	14.4
2	13.5	ZnBr_2_	2.1	9.2
3	13.5	AlCl_3_	3.8	9.8
4	13.5	FeCl_2_	3.8	5.2
5	13.5	MgCl_2_	1.1	12.7
6	13.5	MgCl_2_	5.1	43.9
7	13.5	ZnCl_2_	0	2.5
8	13.5	ZnCl_2_	0.8	3.6
9	13.5	ZnCl_2_	1.2	7.1
10	13.5	ZnCl_2_	2	10
11	13.5	ZnCl_2_	4	16.9
12	13.5	ZnCl_2_	7.1	13.7
13	13.5	ZnCl_2_	17.5	6.9
14	13.5	ZnCl_2_	17.5	10.6
15	8.1	ZnCl_2_	0.7	2.7
16	8.1	ZnCl_2_	17.8	7.3
17	13.5	ZnCl_2_	18	7.1
18	18.9	ZnCl_2_	0.7	7.7
19	18.9	ZnCl_2_	17.4	17.6
20	24.3	ZnCl_2_	17.5	41.3
21	27	ZnCl_2_	0.8	16.2
22	35.1	ZnCl_2_	0.8	37.8

### NMR spectra processing

The NMR pre-processing was adapted from literature.^42^ The entire data analysis process was performed using R (4.3.1).^49^ The R packages from the tidyverse family were utilized to organize the data and create the figures.^50^ First the NMR FID was drift corrected by using 5% of the data points recorded at the end of the acquisition period to calculate a mean value, which was subtracted from all FID values. The *k*th data point from the FID was multiplied with an exponential apodization function featuring a frequency of *W* = 1 Hz.

1 e^−π·*W*·*k*·Δ*t*^

Subsequently, the FID was zero filled by adding 32 768 zeros to the end of the FID resulting in 65 536 data points, which enables the extraction of all recorded information according to the Nyquist–Shannon sampling theorem.^51–54^ Finally, the complex conjugate of the processed FID was Fourier transformed using a Fast Fourier Transformation algorithm.^54^ The resulting NMR spectrum was phase corrected using the phase correction angles *φ*_0_ and *φ*_1_ as well as the following equations.2
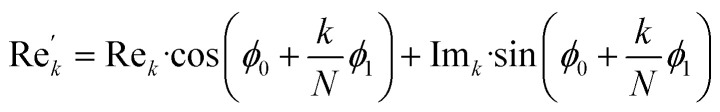
3
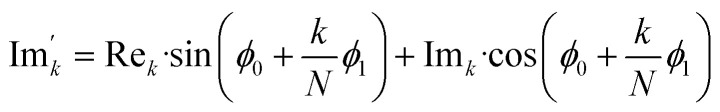
where *k* = 0, …, *N* − 1; Re_*k*_ and Im_*k*_ are the real and imaginary components of the *k*th data point, Re_*k*_′ and Im_*k*_′ are the new components after correction and *N* is the total number of points. *φ*_0_ and *φ*_1_ were manual determined for each measurement series using the graphical user interface of MestReNova (14.2.3–29241).^55^ Finally, the NMR spectra were referenced to the OME methyl signal (3.67 ppm), were cropped to the region of interest between −20 and 14 ppm and the frequency axis as well as all spectra were interpolated to 0.001 ppm intervals. Afterwards, the spectra were normalized to the area of the OME methyl signal (3.39 to 4.00 ppm). The preprocessed NMR spectra were analyzed by integrating the respective signals (OME_1_: 4.70 to 4.96; OME_2+_: 4.96 to 5.14; OME_3+_: 5.14 to 5.26; TRI: 5.33 to 5.62 ppm). Eqn (4)–(6) were used to transform the signal areas (*A*_X_) into concentrations (*c*_X_).4
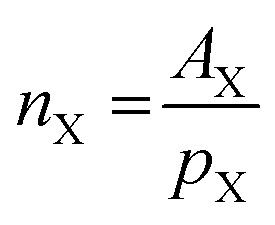
5
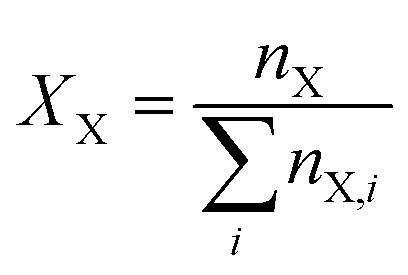
6
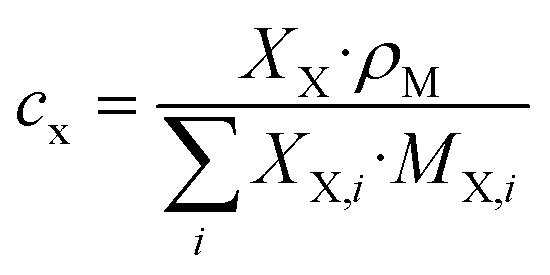
where *n*_X_ is the relative number of molecules, *p*_X_ the respective proton count, *X*_X_ the molar ratio, *ρ*_M_ the density of the reaction mixture and *M*_X,i_ is the molecular mass of the *i*th species. OME_2+_ was calculated based following eqn (7) with the signal areas of OME_2+_ and OME_3+_7
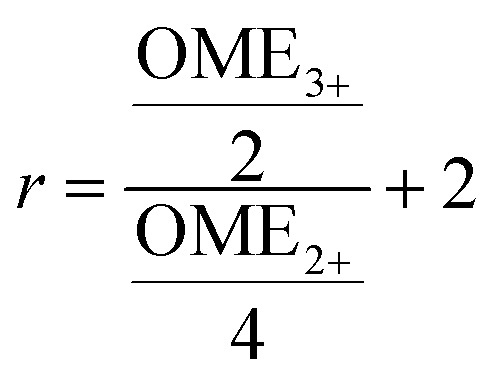


### Kinetic analysis

The R packages FME and deSolve were used to solve the kinetic differential eqn (8)–(12).^55^ To fit the rate constants *k*_1_–*k*_5_ to the experimentally determined concentrations a cost function was defined which aimed to minimize the sum of squared residuals. The experimental and fitted OME_1_ and TRI concentrations could be compared directly while the experimental OME_2+_ concentration was compared to the sum of the fitted OME_2_, OME_3_, OME_4_ and OME_5_ concentrations.8
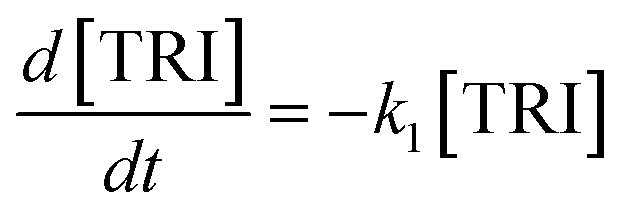
9
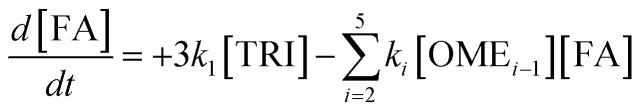
10
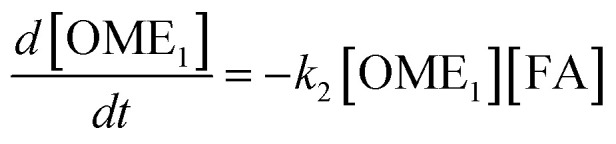
11
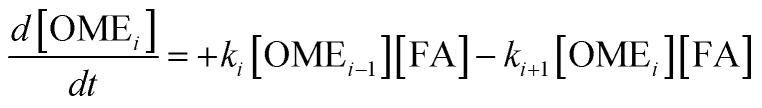
12
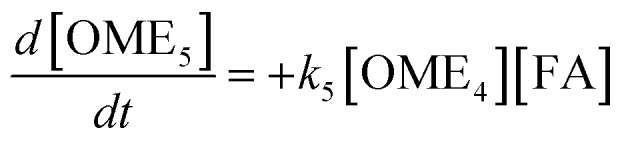


## Conclusions

The influence of the combination of Lewis and Brønsted acids was investigated in detail for the homogeneous catalyzed oxymethylene ether synthesis starting from trioxane and dimethoxymethane. The reaction was monitored by an inline NMR measurement setup. As Brønsted acid (BA) triflic acid was used and different metal halides (FeCl_2_, ZnCl_2_, ZnBr_2_, AlCl_3_ and MgCl_2_) were tested as Lewis-acids (LA). Furthermore, the influence of the variation of the concentration of both, LA and BA, was investigated to gain further insights in the synergetic effects. These experiments have led to several key results:

• The LA is unactive without the presence of BA under the investigated conditions.

• The increase of Lewis acidity results in a lower reaction rate.

• The increase of BA concentration at a constant LA amount leads to an exponential increase of the reaction rate.

• The increase of LA concentration at a constant BA amount leads to an increase up to a BA–LA-ratio of 1 : 3 and a subsequent decrease of the reaction rate at a lower BA–LA-ratio.

Based on these results the importance of the electron density within the formaldehyde intermediate was concluded to be a crucial factor for the catalytic activity of the LA. By interaction of the LA with the oxygen atom of the FA molecule, a partial positive charge is generated at the carbon atom which enhances the reaction rate. On the contrary the electron withdrawing effect of the LA reduces the electron density at the oxygen atom which decreases the reaction rate. Within this study MgCl_2_, the weakest investigated LA based on the Pauling electronegativity, already resulted in the strongest increase of the reaction rate, revealing the dominant influence of the electron density at the oxygen atom on the catalytic activity. The decrease of the reaction rate at BA–LA-ratios lower than 1 : 3 was proposed to be caused by the higher probability of more than one LA interacting with the FA and, thus, leading to the same effect of reduced electron density at the oxygen atom. If the BA–LA-ratio of about three, being the optimum ratio in terms of reaction rates, is universal for this reaction system or depends on the reactivity ratios of the both catalysts, needs to be addressed in further studies.

## Conflicts of interest

There are no conflicts to declare.

## Supplementary Material

RA-014-D4RA00744A-s001
