# Supplementary material for: Investigation of the cooperative-effects of Lewis- and Brønstedt acids in homogeneously catalyzed OME fuel synthesis by inline-NMR monitoring
Source: RSC Adv. 2024 May 8;14(21):14942–8. doi: 10.1039/d4ra00744a (PMC11078091; doi:10.1039/d4ra00744a)
Supplement: RA-014-D4RA00744A-s001 [file RA-014-D4RA00744A-s001.pdf]

Electronic Supplementary Material (ESI) for RSC Advances.
This journal is © The Royal Society of Chemistry 2024

Investigation of the cooperative-effects of Lewis- and Brønstedt acids in homogeneously catalyzed OME fuel synthesis by inline-NMR monitoring

-

Supporting Information

Patrick Endres,[a,b] Timo Schuett,[a,b] Stefan Zechel,[a,b] Martin D. Hager,[a,b,c,d] Robert Geitner*[e] and Ulrich S. Schubert*[a,b,c,d]

a Patrick Endres, Timo Schuett, Julian Kimmig, Dr. Stefan Zechel, Dr. Martin D. Hager, Prof. Dr. Ulrich S. Schubert
Laboratory of Organic and Macromolecular Chemistry (IOMC),
Friedrich Schiller University Jena,
Humboldtstr. 10, 07743 Jena, Germany

b Patrick Endres, Timo Schuett, Julian Kimmig, Dr. Stefan Zechel, Dr. Martin D. Hager, Prof. Dr. Ulrich S. Schubert
Jena Center for Soft Matter (JCSM),
Friedrich Schiller University Jena,
Philosophenweg 7,
07743 Jena, Germany

c Dr. Martin D. Hager, Prof. Dr. Ulrich S. Schubert
Center for Energy and Environmental Chemistry Jena (CEEC Jena),
Friedrich Schiller University Jena,
Philosophenweg 7a, 07743 Jena, Germany
E-mail: ulrich.schubert@uni-jena.de

d Dr. Martin D. Hager, Prof. Dr. Ulrich S. Schubert
Helmholtz Insitute for Polymers in Energy Applications Jena (HIPOLE Jena),
Lessingstraße 12-14, 07743 Jena, Germany

e Jun.-Prof. Dr. Robert Geitner
Institute for Chemistry and Bioengineering
Technical University Ilmenau
Weimarer Str. 32, 98693 Ilmenau, Germany
E-mail: robert.geitner@tu-ilmenau.de

Table of contents

1. NMR measurement cycle S1
2. NMR measurements at selected reaction times S2
3. Kinetic fitting of molar share over time S3

1. NMR measurement cycle

The NMR measurements were started with an initial shim on sample, and then ^{1}H NMR spectra were acquired. This process was repeated ten times before conducting another shim on sample. This entire cycle of measurements and shimming was repeated up to a maximum of 50 times. (Fig. S1)

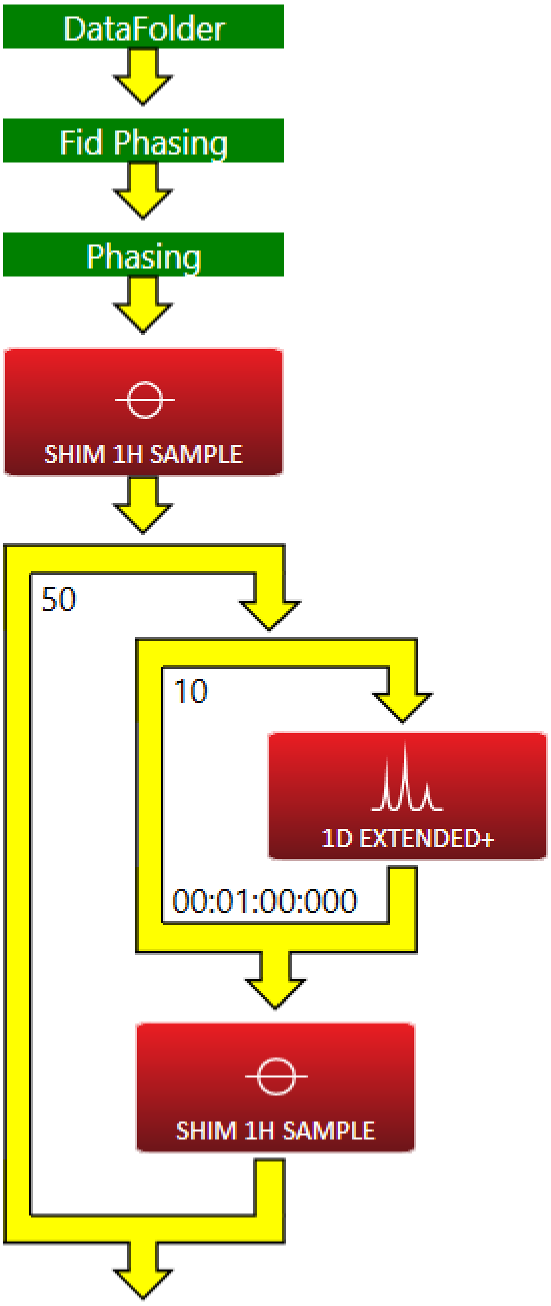

Figure S1: Analytic cycle of the online NMR measurements.

2. ^{1}H NMR measurements at selected reaction times

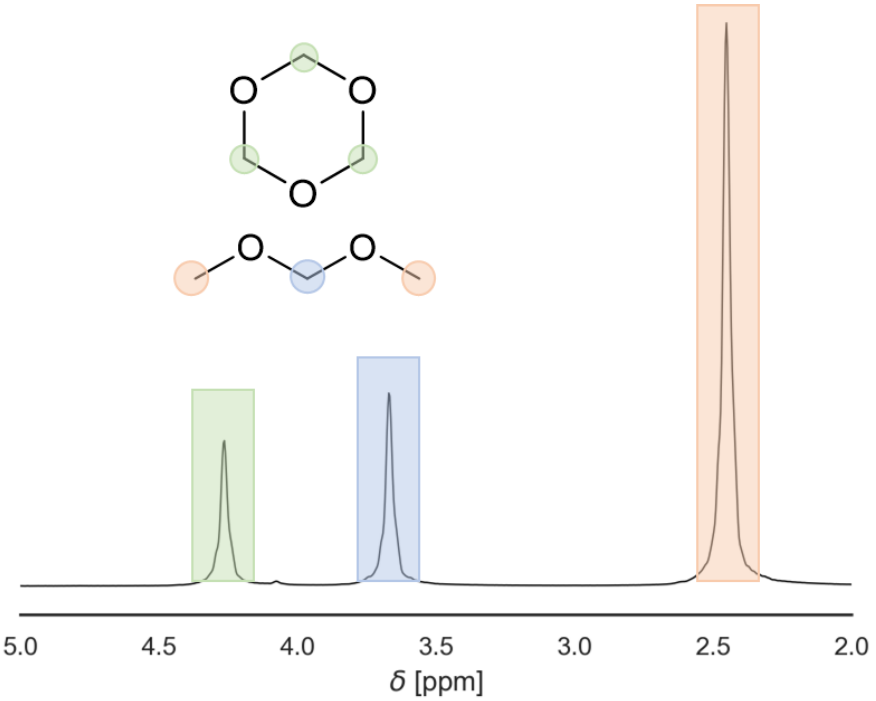

Figure S2: ^{1}H NMR spectrum after reactor loading, dimethoxy methane 150 g, trioxane 44.4 g, $ZnCl_2$ 17.5 mmol L^{-1}, 20 °C (43 MHz).

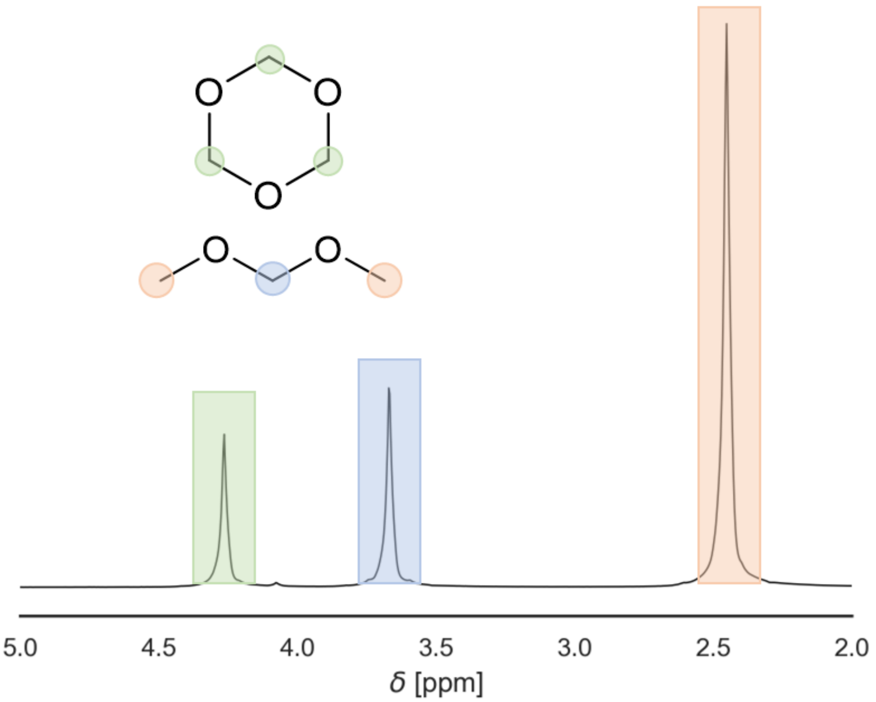

Figure 3: ^{1}H NMR spectrum after 20 min prior to the addition of the Brønsted acid catalyst, dimethoxy methane 150 g, trioxane 44.4 g, $ZnCl_2$ 17.5 mmol L^{-1}, 20 °C (43 MHz).

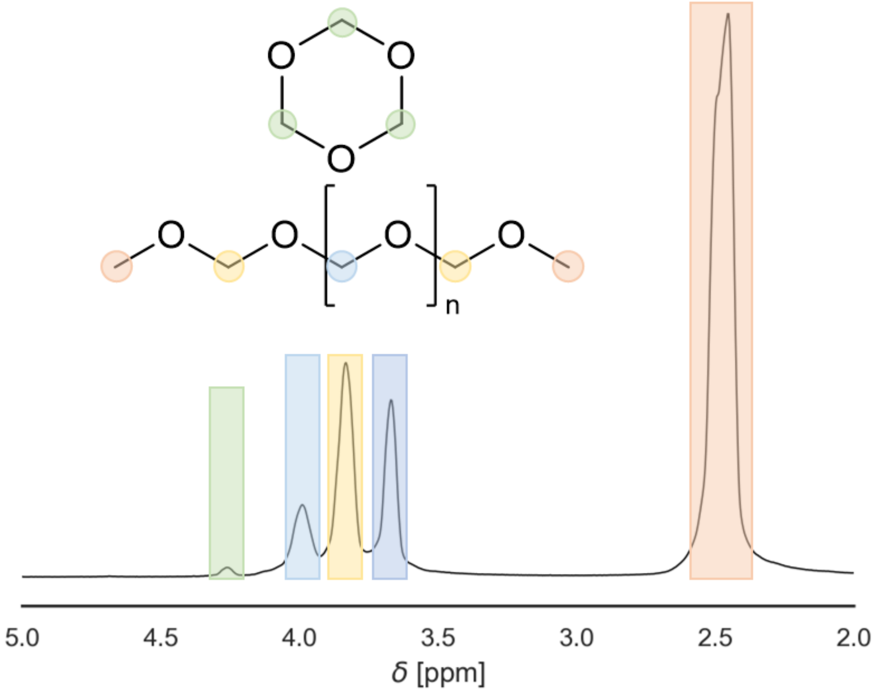

Figure 4: ^{1}H NMR spectrum after 120 min at equilibrium conditions, dimethoxy methane 150 g, trioxane 44.4 g, triflic acid 13.5 mmol L^{-1}, $ZnCl_2$ 17.5 mmol L^{-1}, 20 °C (43 MHz).

3. Kinetic fitting of molar share over time

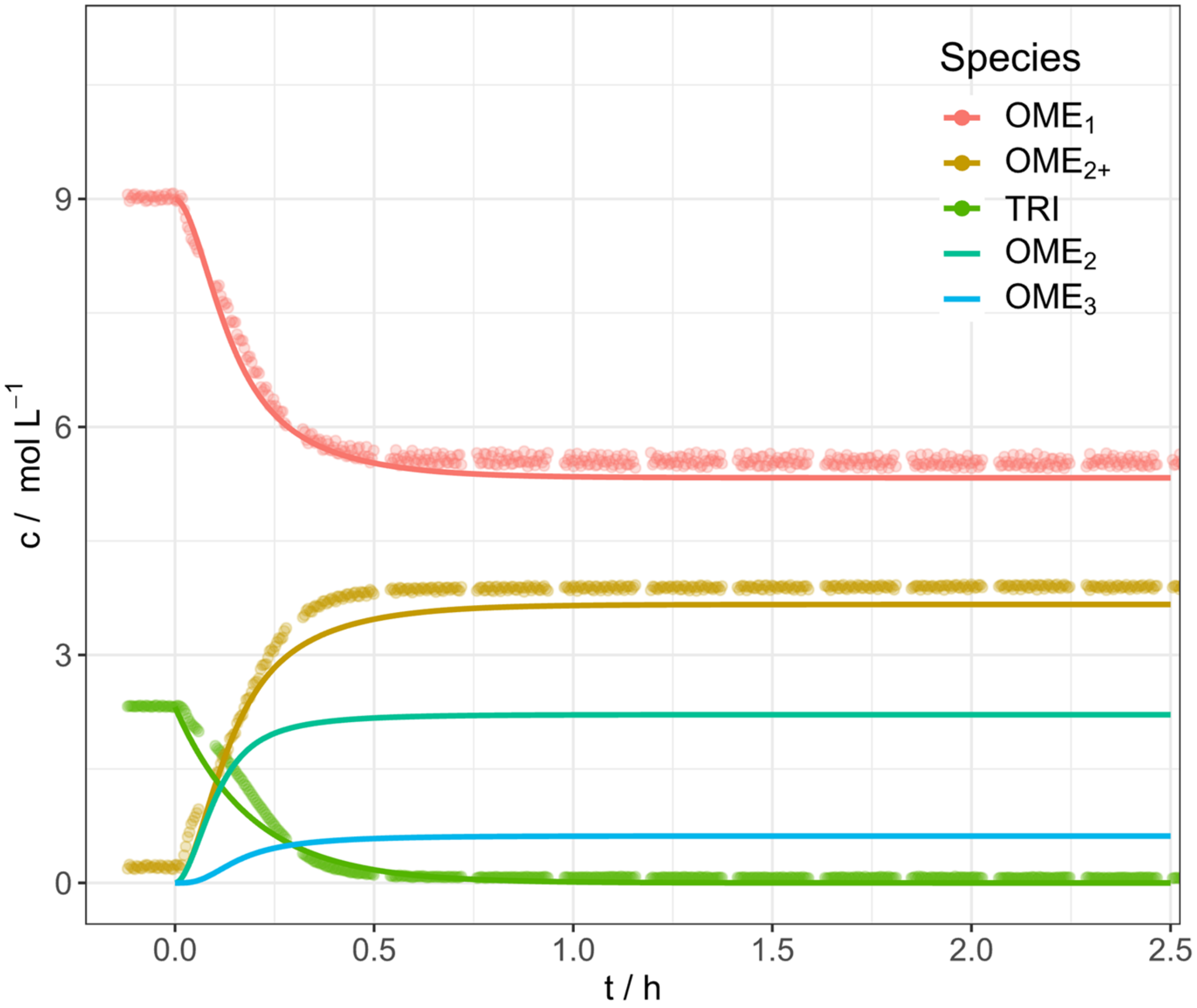

Figure 6: Experimentally extracted concentrations of OME_x over time with kinetic fits, dimethoxy methane 150 g, trioxane 44.4 g, triflic acid 13.5 mmol L^{-1}, $ZnCl_2$ 2.9 mmol L^{-1}, 20 °C.
